# Greenhouse Gas Emissions and the Australian Diet—Comparing Dietary Recommendations with Average Intakes

**DOI:** 10.3390/nu6010289

**Published:** 2014-01-08

**Authors:** Gilly A. Hendrie, Brad G. Ridoutt, Thomas O. Wiedmann, Manny Noakes

**Affiliations:** 1 Animal, Food and Health Sciences, Commonwealth Scientific Industrial Research Organisation (CSIRO), P.O. BOX 10041, Adelaide 5000, Australia; E-Mail: manny.noakes@csiro.au; 2 Sustainable Agricultural Flagship, Commonwealth Scientific Industrial Research Organisation (CSIRO), Bayview Avenue, Clayton South, Victoria 3169, Australia; E-Mail: brad.ridoutt@csiro.au; 3 School of Civil and Environmental Engineering, The University of New South Wales, Sydney 2052, Australia; E-Mail: t.wiedmann@unsw.edu.au; 4 Ecosystem Sciences, Commonwealth Scientific Industrial Research Organisation (CSIRO), Canberra 2600, Australia

**Keywords:** diet, sustainability, environmental impacts, food security, nutrient security, greenhouse gas emissions

## Abstract

Nutrition guidelines now consider the environmental impact of food choices as well as maintaining health. In Australia there is insufficient data quantifying the environmental impact of diets, limiting our ability to make evidence-based recommendations. This paper used an environmentally extended input-output model of the economy to estimate greenhouse gas emissions (GHGe) for different food sectors. These data were augmented with food intake estimates from the 1995 Australian National Nutrition Survey. The GHGe of the average Australian diet was 14.5 kg carbon dioxide equivalents (CO_2_e) per person per day. The recommended dietary patterns in the Australian Dietary Guidelines are nutrient rich and have the lowest GHGe (~25% lower than the average diet). Food groups that made the greatest contribution to diet-related GHGe were red meat (8.0 kg CO_2_e per person per day) and energy-dense, nutrient poor “non-core” foods (3.9 kg CO_2_e). Non-core foods accounted for 27% of the diet-related emissions. A reduction in non-core foods and consuming the recommended serves of core foods are strategies which may achieve benefits for population health and the environment. These data will enable comparisons between changes in dietary intake and GHGe over time, and provide a reference point for diets which meet population nutrient requirements and have the lowest GHGe.

## 1. Introduction

Promoting good nutrition and addressing the escalating costs of health care associated with obesity and other chronic conditions is a major challenge faced by societies and their governments. Equally challenging is addressing the issue of environmental sustainability of our food supply and consequences for food and nutrition security. This concern for a sustainable food supply and the urgency to reduce atmospheric carbon to avoid climate change demands informed behaviour changes to reduce diet-related greenhouse gas emissions (GHGe).

The production and consumption of food has a range of environmental consequences [1,2,3,4,5]. Nutrition guidelines are now increasingly focusing some attention on the environmental impact of food choices as well as the traditional nutritional metrics that relate to meeting nutrient requirements, promoting health and averting chronic disease [6]. However, we do not have sufficient data quantifying the environmental impact of dietary patterns on which to base these recommendations.

GHGe through their impact on climate change, challenge long term food security but also have implications for human health and ecosystems. GHGe is only one measure of environmental impact. The most common greenhouse gases are carbon dioxide (CO_2_), methane (CH_4_), nitrous oxide (N_2_O), hydrofluorocarbons (HFC), perfluorocarbons (PFC) and sulphur hexafluoride (SF_6_). Many countries report emissions from these gases under the Kyoto Protocol to the United Nations Framework Convention on Climate Change [7]. The emissions are aggregated into carbon dioxide equivalents (CO_2_e) adjusting for each gas’s global warming potential. The Australian government monitors GHGe through the National Greenhouse Gas Inventory according to defined sectors, including agriculture which accounts for 16% of Australia’s emissions [8].

Research conducted in the United Kingdom has shown that changing population food choices to meet dietary requirements could help towards mitigating climate change [9]. However in Europe, modelling changes towards a healthier diet resulted in minimal reductions in environmental impacts [1]. Within the French context, self-selected diets of the highest nutritional quality were not those with the lowest diet-related GHGe. In particular, the consumption of sweets and salted snacks foods were negatively correlated with GHGe, meaning they have low GHGe [10]. Therefore, proposing dietary changes should start from a health perspective because not all diets that meet the dietary requirements for health will necessarily have lower GHGe.

It has also been suggested that overweight and obesity has implications for the environment. Compared with a normal weight population, a population with 40% obesity requires 19% more energy from food [11]. Therefore, whilst GHGe estimates of isocaloric dietary comparisons can provide useful information on their relative impact, limiting excessive energy consumption as a strategy to reduce GHGe also warrants important consideration. Energy reduction as a strategy has clear health benefits for obesity, type 2 diabetes and other chronic disease reduction as well as environmental benefits.

No government has been successful in limiting excessive food intake, although some have made progress in getting populations to change their food choices. Food is fundamental to our health and it is important to understand the various components that influence a population’s food choices. Australia’s Food and Nutrition Policy was developed to facilitate and support action towards healthier food choices through the entire food and nutrition system [12]. This system includes the food supply (including imports, exports, food production, processing and distribution); consumers’ food selection and consumption (including price, knowledge, education and preferences); as well as the overarching policy environment. Governments can introduce policy measures that affect the food system and food choices including introduction of taxes or levies that may impact on price [13], regulations on the sale or purchase of products [14], provide support for product development and reformulation [15], intitiate monitoring and surveillance mechanisms [12], and develop strategies which relate to education and communication to consumers [6]. A sound knowledge of the food and nutrition system is crucial to respond to future challenges such as food and environmental sustainability, and chronic diseases such as obesity [12].

Understanding the optimal dietary patterns that promote health, reduce the risk of chronic diseases, and lower GHGe profiles may help inform government food policy [16]. Currently in Australia there is limited evidence regarding the environmental impacts of the population’s diet. Understanding GHGe associated with both current and recommended dietary intakes may inform dietary choices which meet both nutrition and environmental goals and are adaptable to cultural norms in eating behaviours. The aim of this modelling exercise was to conduct an assessment of the GHGe from the average Australian diet as at 1995 and compare these data with recommended diets which meet current dietary guidelines. This work will also enable future comparisons of changes in dietary intake over time on GHGe.

## 2. Experimental Section

### 2.1. Modelling of Greenhouse Gas Emissions

Greenhouse gas emissions (GHGe) for the average Australian adult diet and alternative dietary scenarios were assessed using an environmentally extended input-output model of the Australian economy. The input-output modelling approach, based on an economy-wide assessment of financial transactions between industrial sectors, was deemed appropriate because of the national scale of the study and its focus upon food categories rather than individual food products. Input-output analysis also has the advantage of providing a complete assessment of GHGe (to the point of purchase), avoiding the truncation errors associated with the application of a system boundary in process life cycle assessment [17,18,19].

For this particular study the AUS-MRIO model was used, which is the latest elaboration of the Australian Multi Regional Input-Output (MRIO) model developed by Lenzen and colleagues [20,21]. At the time of use, AUS-MRIO characterised the Australian economy in the financial year 2007/2008 and provided resolution to 344 sectors. The main data sources are the Australian national input-output tables [22,23] and national greenhouse gas inventory [8]. Six major (CO_2_, CH_4_, N_2_O, HFC, PFC, SF_6_) and four minor (NMVOC (Non-methane volatile organic compounds), NO*x*, SO_2_, CO) greenhouse gases are included in the model. Results are reported in CO_2_e utilizing the 100-year global warming potentials published in the Intergovernmental Panel on Climate Change (IPCC) Second Assessment Report as used in the Kyoto Protocol. Further detail of the model development is described elsewhere [24].

Due to the expected important contribution of certain fresh meat and meat products to dietary GHGe [25,26] additional sector breakdown was performed for these important food categories. Life cycle GHGe factors for fresh meat and meat products were disaggregated into factors for fresh meat and meat products from beef cattle, sheep and pigs by running the model three times with only one type of animal product being used as input from animal production to fresh meat and meat products. For example, to derive the life cycle GHGe factor for beef it was assumed that the fresh meat and meat product sectors bought all of their animal products only from the beef cattle sector.

Imports to and exports from Australia are represented with a “rest-of-world” region in the AUS-MRIO model. Therefore, GHGe associated with any good or service imported to Australia from abroad are accounted for in the model, including all inputs to food production. However, there is no differentiation by country of origin. In terms of economic value, between 3% and 6% of agricultural products and between 1% and 11% of food products are imported from outside of Australia [27].

### 2.2. Dietary Intake Data

Dietary data for the Australian population were augmented with the environmental input-output model. The dietary intake data is from the most recent National survey of Australia adults—the 1995 Australian National Nutrition Survey. The self-report population survey collected data from a representative sample of the Australian population and the method is described in detail elsewhere [28]. Using the intake data for adults only (aged 19 years and older) and applying the population weightings provided, an average daily intake per food sector per person was calculated. Dietary intake is expressed in grams consumed per person per day.

Individual foods were assigned into food sectors consistent with the input-output model. Mixed dishes, including combinations of foods like casseroles and stews, were identified and disaggregated into their respective food sectors (based on percentages used in previous research [29]). For example, mixed dishes such as a meat dish with a cereal product (e.g., beef stew with rice, chicken with pasta), was assumed to be 35% meat, 50% cereal, 5% sauce and 10% oil. The meat component was assigned to the stated meat type (beef, lamb, pork, chicken or fish) and the cereal component assigned to pasta and rice in a 50:50 proportion by weight. Sandwiches were also disaggregated back to bread and fillings.

To convert between raw and cooked products in the input-output model and food consumption data, adjustment factors were applied to account for loss or gain of moisture during the cooking process. These adjustment factors were applied without impacting on nutrient profiles. Cooked meat to raw meat was multiplied by a factor of 1.4 and cooked rice back to raw rice by dividing by a factor of 3 [30].

To describe the diet scenarios, food sector groups were assigned to commonly used food groupings, as consistent as possible with the Australian Dietary Guidelines [6]. Core foods groups included red meat (beef and lamb, pork), chicken, fish, eggs, breads and cereals, fruit, vegetables, dairy foods and unsaturated oils and spreads. Other food groups (non-core) included snack foods (such as pies, cakes, biscuits, chips, high fat dairy desserts and confectionery); soft drinks, tea, coffee, sugar and miscellaneous foods; processed meats (such as sausages and salami); saturated fats and oils; and alcohol.

To allow for the dietary data to be merged with the GHGe data, an average purchase cost was applied to each food sector and an adjustment factor applied to convert from purchased price back to basic price [27]. The average purchase cost of each food sector was calculated using online data from a leading supermarket chain (conducted on 17 September 2012). A representative price was assigned to each food group within the food sector, and the average cost per gram per food sector calculated. The global warming potential (expressed as kilograms CO_2_e) of the average diet and recommended diet scenarios were calculated by multiplying the weighted average cost per food sector with its GHGe intensity derived from input-output calculations (Equations 1–7, a worked example calculation to estimate the greenhouse gas emissions (in CO_2_ equivalents) from the confectionery food group):

Average consumption of confectionery per person per day = 11 g
(1)

Estimated cost of confectionery is $14.75/kg = $0.01475/g
(2)

Cost of confectionery consumed = 11 × 0.01475 = $0.16
(3)

AUS MRIO model estimated kg CO_2_ = 0.411kg CO_2_e per $ confectionery consumed (4)

Estimated kg CO_2_e from confectionary = 0.411 × 0.16 = 0.066 kg CO_2_e (5)

Adjustment factor purchase to basic price for Sugar and Confectionery manufacturing = 1.5795
(6)

Estimated kg CO_2_e for confectionery in average Australian diet = 0.066/1.5795 = 0.042 kg CO_2_e (7)

Dietary modelling examined the impacts of proposed dietary patterns on the GHGe of the diet and the relative impact on the overall nutrient profiles of these diets. The modelling was based on a few scenarios: the average diet as assessed in 1995; reducing non-core foods in the average diet; and eating a recommended diet. The amount of each food group included in the scenarios is listed in Table 1. In summary, the following scenarios were modelled:

(1) Average diet: average Australian diet based on the foods and quantities reported in the 1995 National Nutrition Survey.

(2) Average diet with minimal non-core foods: Similar foods and quantities as the average diet with minimal inclusion of energy-dense, processed non-core foods. This scenario excludes processed meat, snack foods, confectionery, soft drinks, saturated fats and oils, and alcohol, however does include tea and coffee and offal meat in the same quantities as the average diet scenario.

(3) Total diet: A recommended dietary pattern consistent with the Australian Dietary Guidelines. It is designed to promote health and wellbeing, and is a flexible eating pattern that includes some non-core, energy-dense foods. The diet is designed to meet the energy and nutrient needs of the population. The food grouping recommendations were based on the average of diets for Australian males (160 cm) and females (150 cm) aged 31–50 years and assumed to be very sedentary (Physical Activity Level (PAL) = 1.4) [31]. The diet recommends one serve of red meat and one serve of alternative protein sources per day. One serve of red meat is equal to 65 g of cooked beef, lamb, veal and pork. For this modelling scenario of one day, the 65 g of cooked meat was assigned as beef and lamb. The alternative meat sources include chicken, fish and eggs. One 100 g serve was assigned as 50% chicken, 30% fish (20% fresh, 10% canned) and 20% eggs. The total diet also includes one and a half serves of non-core foods which were assumed to be snack foods (assigned as 50% sweet biscuits and 50% potato crisps by weight).

(4) Foundation diet: A recommended dietary pattern that meets the minimum nutrient and energy requirements for the population. It is designed to be an energy constrained diet that includes only core foods (in similar amounts to the total diet).

The nutrient adequacy of each diet scenario was compared to the recommended daily intake (RDI) values for Australians [32]. The average RDI for men and women aged 31–50 years was used as the reference value. Diet scenario adequacy is presented as a percentage of the reference value.

**Table 1:** Summary of diets and greenhouse gas emissions (GHGe) ^1^ for food groups for the diet scenarios ^2^, as an average consumption per person per day.

Core Food Groups	Average Diet (9409 kJ)		Average Diet Scenario		Recommended Diet Scenarios			
			Minimal Non-Core Foods (6188 kJ)		Total Diet (9398 kJ)		Foundation Diet (8629 kJ)	
	Grams	GHGe (kg CO_2_e)	Grams	GHGe (kg CO_2_e)	Grams	GHGe (kg CO_2_e)	Grams	GHGe (kg CO_2_e)
Red meat	73	8.04	73	8.04	65	7.83	65	7.83
Poultry	35	0.24	35	0.24	50	0.33	50	0.33
Fish	24	0.12	24	0.12	30	0.22	30	0.22
Eggs	14	0.02	14	0.02	8	0.01	8	0.01
Breads and cereals	244	0.63	244	0.63	324	0.76	324	0.76
Fruit	210	0.28	210	0.28	300	0.17	300	0.17
Vegetables	331	0.46	331	0.46	432	0.33	432	0.33
Dairy	263	0.72	263	0.72	408	1.12	408	1.12
Unsaturated oils and spreads	16	0.05	16	0.05	26	0.09	26	0.09
*Non-Core Foods*								
Snack foods	172	1.12	0	0	49	0.2	0	0
Processed meats	27	1.76	1	0.07	0	0	0	0
Sugar, tea, coffee, miscellaneous	298	0.58	29	0.41	0	0	0	0
Alcohol	254	0.44	0	0	0	0	0	0
Saturated fats and oils	4	0.01	0	0	0	0	0	0
Total diet-GHGe (kg CO_2_e)	14.5		-	11.0	-	11.1	-	10.9
% diet-GHGe from core foods	72.9		-	95.6	-	97.8	-	100.0
% diet-GHGe from non-core foods	27.1		-	4.4	-	2.2	-	0.0

^1^ GHGe expressed as kilograms carbon dioxide equivalents (kg CO_2_e); ^2^ Summary of diet scenarios: (1) Average diet: is the average Australian adult diet based on the 1995 National Nutrition Survey; (2) Minimal non-core foods: is the average diet with most non-core foods removed. This scenario includes a small amount of tea and coffee and offal meat (same as the average diet) but does not include snack foods, sugar and confectionery, alcohol and saturated fats and oils; (3) Total diet: is based on the Australian Dietary Guidelines and represents the average recommendation for male and females aged 31–50 years, includes a small about of non-core foods; (4) Foundation diet: is similar to the Total diet without non-core foods.

## 3. Results

**Figure 1:**
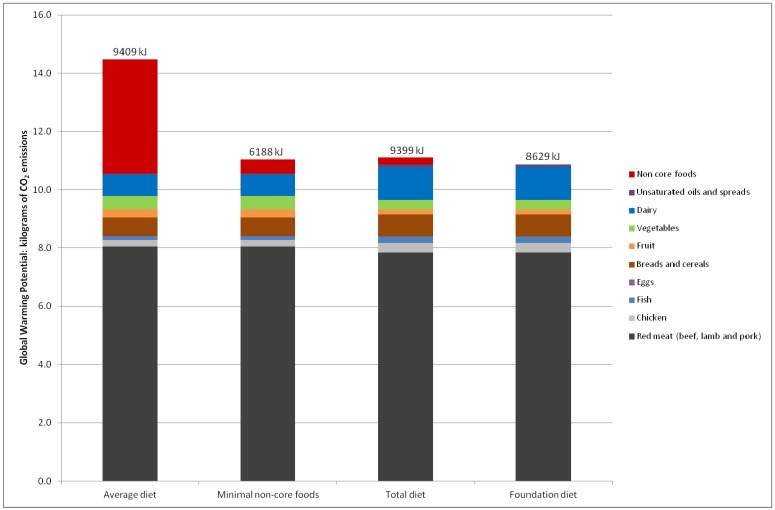
Greenhouse Gas Emissions in kilograms carbon dioxide equivalents (kg CO_2_e) per person per day for the diet scenarios.

The average Australian diet as assessed in 1995 had the highest GHGe at 14.5 kg CO_2_e per person per day and was also highest in kilojoules (~9400 kJ, Table 1). The Foundation diet had the lowest emissions—about 25% lower than the average Australian diet (10.9 kg CO_2_e per person per day). The estimated emissions from the recommended Total diet and the average diet without non-core foods were similar, despite the recommended diet providing more energy (9398 *vs.* 6188 kJ, Figure 1).

The food groups that made the greatest contribution to the diet-related GHGe in the 1995 average Australian diet were red meat (8.0 kg CO_2_e per person per day) and non-core foods (3.9 kg CO_2_e per person per day). Non-core foods accounted for 27% of the emissions from the average Australian diet (Table 1).

The macronutrient profiles of the diet scenarios were relatively similar with carbohydrates contributing about 45% of energy, protein between 16% and 20% and fat about 35% of total energy (Table 2). From a micronutrient adequacy point of view, the recommended total and foundation diets are designed to be nutritionally adequate and meet the suggested dietary targets for all nutrients. On the contrary, the average diet does not meet the suggested dietary target for dietary fibre, vitamin A, folate, calcium and magnesium. However, when most non-core foods were removed from the average diet, nutrient intake was further compromised (Table 2).

In summary, at a population level, the recommended Total and Foundation diets are nutrient rich and have the lowest GHGe.

**Table 2:** Macro and micronutrient adequacy ^1^ of the diet scenarios, expressed as a percentage of total energy or a percentage of the dietary target.

Diet Composition		Average Diet	Average Diet Scenario: Minimal Non-Core Foods
*Macronutrients*		*% of Energy*	
Energy (kJ)		9409	6188
Carbohydrate		46	45
Protein		16	20
Fat		34	35
Saturated fat		13	12
*Micronutrients*	*RDI/AI*	*% RDI/AI*	
Dietary fibre	27.5 g	83	71
Vitamin A	800 μg	79	61
Folate	400 μg	67	58
Calcium	1125 mg	76	63
Magnesium	366 mg	90	73
Zinc	11 mg	113	94
Potassium	3300 mg	97	82

^1^ Mean recommended dietary intake (RDI)/Adequate intake (AI) for men and women aged 19–50 years was used as the target. AI was used for dietary fibre and potassium. For nutrients not presented 100% of RDI was met on all diet scenarios.

## 4. Discussion

This study modelled the GHGe from the Australian diet and compared this to emissions from the recommended dietary patterns. We have shown that on average, the GHGe of the recommended dietary patterns for Australians are about 25% lower than the average diet. We have also shown synergies between the nutrition and climate impacts of recommendations which advocate a reduction in non-core food consumption and realignment of dietary choices to better conform to Australian Dietary Guidelines.

Dietary recommendations are turning towards dietary patterns that are healthy but also sustainable and more favorable to the environment [33]. Until now there has been little evidence to quantify what this means within the Australian context. These data serve as a benchmark to track changes in GHGe from the diet over time as well as provide a reference point for diets which meet both the nutritional needs of the population and have the lowest GHGe.

The key food differences between the average Australian diet and the recommended dietary patterns are the need to eat more fruit, vegetables and legumes, and dairy. The recommended dietary patterns also require eating less food and drinks high in saturated fat, added sugar, salt, or alcohol—referred to as non-core foods. Non-core foods contribute about 27% of total GHGe in the average Australian diet; however they have been shown to contribute 36% of total kilojoules for adults [34]. Non-core foods as a group are often described as being energy dense but nutrient poor because they provide significant amounts of kilojoules relative to the nutrients they contain. While non-core food might be energy dense relative to healthy “core” foods, our study suggests that their “emissions density” can vary greatly. GHGe associated with alcohol consumption is relatively low compared to the greatest contributors within the non-core category-processed meat and snack foods. However, to focus purely on reducing GHGe in this way conflicts with a health promoting dietary pattern consistent with the Australian Dietary Guidelines. But it does highlight the importance of ongoing efforts by food producers in Australia to adopt more sustainable production practices which minimise GHG impact as well as other environmental impacts, including water, land and biodiversity.

The primary focus of this paper was to estimate the GHGe of recommended dietary patterns. Modelling the GHGe of the dietary pattern scenarios behind the Australian Dietary Guidelines ensures that health outcomes are at the forefront of our focus. In Australia, the recommended dietary pattern has been extensively modelled for nutrition adequacy [35] and forms the basis of public health nutrition advice. The Total diet pattern provides a flexible eating pattern while still focused on health and wellbeing. This pattern of eating is lower in energy and about 23% lower in GHGe than the average Australian diet. A similar reduction in GHGe would result if Australians removed most non-core, energy-dense foods from their current pattern of eating, however this would also reduce total kilojoule intake by over a third. Because total kilojoule consumption has been shown to be positively correlated with diet-related GHGe [10], it is important to compare isocaloric scenarios when exploring the environmental impacts of diet.

Government public health policies and regulations influence the food and nutrition system, and ultimately influences what people eat [12]. Government initiatives that address overconsumption generally will have co-benefits for health and the environment. Reduced energy intake will reduce the risk of obesity, which in turn is related to lower GHGes. The Foundation diet pattern is designed to be energy constrained, nutritionally complete and has 25% lower emissions than the average diet. However, the population may not accept the complete absence of non-core or “treat” foods. An alternative strategy to address obesity is to maintain the current eating pattern but reduce intake of all foods by 10%, for example. This would reduce energy intake and total GHGe by 10%. While this scenario may be more acceptable to the population, the resulting diet would not be nutritionally complete. Therefore based on the scenarios examined in this modelling exercise, the dietary patterns recommended by the Australian Dietary Guidelines are the optimal scenarios for balancing nutrition adequacy and reducing diet-related GHGe.

Red meat is the greatest contributor to diet-related emissions (which is also true in other countries [1,2,4,5,36,37] and livestock a major contributor to agricultural emissions in general [3,38]. Suggestions to reduce red meat intake as a sole strategy, poses nutritional challenges for some key nutrients such as highly bioavailable iron, zinc, and long chain ω-3 fats, as red meat is an important source of these nutrients in the Australian diet [29,39]. Scarborough *et al*. modified the amount and sources of meat in their GHGe modelling of the UK diet. A scenario which reduced all livestock products by 50%, including meat and dairy, resulted in a 19% reduction in GHGe but also a reduction in vitamin A, zinc and dietary calcium compared to the average UK diet [37]. This is further highlighted by the modelling undertaken to guide the Australian Dietary Guidelines which found that 455 g of red meat per week is not a limit but a recommendation to meet estimated nutrient needs [31].

Replacing red meat with alternative protein sources, including vegetarian alternatives, is another strategy to reduce diet-related GHGe. Scarborough *et al*. reported that maintaining the amount of meat but shifting from red meat to white meat resulted in a 9% reduction in GHGe but vitamin A and zinc were lower than the average diet [37]. Others have reported that replacing all meat with dairy foods in an isocaloric scenario decreases GHGe by 22% but also decreases protein and increases fat intake relative to the average omnivore diet it replaces [4]. Whereas modelling the environmental impacts of changes towards healthier diets in Europe suggests that the positive effects of small reductions in meat are cancelled out by increased fish, cereal and vegetable intakes, resulting is no overall environmental benefits [1]. Our concern is that simplistic recommendations fail to consider the full nutritional consequences of such a change, as well as the full range of environmental burdens associated with the production of the alternative protein sources—especially environmental impacts related to land and water use, and indirect land use change. In addition, consumer acceptance of dietary transformations, including modifications to protein choices and plant-based diets [40], needs to be considered when making such recommendations. These modelling scenarios from Australia, UK and Europe highlight how challenging it is to balance nutrition adequacy and environmental impacts.

It is worth noting that the data for GHGe are estimates which may vary widely within food sectors. This is because food production systems vary, especially in Australia which is such a large continent with diverse climatic and environmental contexts. This leads to considerable variation in GHGe for every type of food. For beef cattle production, GHGe vary by production systems and by region, making it difficult to provide a single estimate. For example, currently available estimates suggest the carbon footprint could be in the order of 10–12 kg of CO_2_e per kg live weight [41] or up to 17.5 kg CO_2_e per kg live weight for equivalent cattle produced [42]. Ongoing research is underway to better understand how to more accurately measure and reduce carbon footprints of meat production and consequently to understand how to better represent this variability.

This variation highlights the potential to reduce diet-related GHGe through improvements in the upstream agricultural sectors [3]. There is some evidence to suggest that the adoption of more efficient production practices has reduced GHGe intensity, and that eliminating food wastage pre and post purchase can also reduce GHGe [4]. Therefore strategies to reduce the environmental impact of our diet do not necessarily have to rely on changes in diet composition and patterns of eating.

Friel *et al.* [43] used existing knowledge of the variation in the environmental impacts within each food group to compile a “healthy and sustainable” food basket. They used existing data from life cycle analysis to replace food items typically consumed by Australians in each of the five food groups with a comparable food item that had a lower GHGe. Non-core foods were not included in this exercise but environmental impacts of the diet were considered more broadly to include GHGe, water use and loss of biodiversity. Here we studied GHGe associated with diet; however, we acknowledge that this is not a complete measure of environmental impact. Water, energy, land use, loss of biodiversity and a range of other impacts must all be considered to fully understand environmentally sustainable food systems [44].

There were significant challenges in augmenting the dietary and GHGe datasets and assumptions at various stages of this modelling exercise need to be considered. Firstly, food groupings within each disciplines database differ but needed to be matched. For example dietary recommendations are made for consumption of starchy, green, brassica and orange vegetables however there is only one broad agricultural food sector—vegetables. The national input-output tables are based on financial flows and not physical flows and price is used to convert between them. Therefore food grouping were assigned a cost per gram to match the input-output model units which were expressed as GHGe per dollar. In Australia we have two dominate supermarket chains, and so average prices were taken from one of these chains at a particular point in time. Due to the GHGe intensity of meat, the model is more sensitive to assumptions about meat prices than other food groups. An average price was taken for all cuts of meat and special deals were avoided. Preliminary sensitivity analysis suggests that if a cheaper purchase price for beef was chosen, for example 15% cheaper, then the estimate for GHGe from the diet goes down by half as much (~7%), because meat contributes about half of the total diet-related GHGe. Other assumptions including the adjustment factors for moisture changes when cooking rice and meat need to be considered as these can vary [45], which will influence the overall GHGe estimates for these food groupings.

It should also be noted that there are different approaches to Life Cycle Assessment (LCA) used to measure GHGe. The input-output LCA approach used in this study uses data from the national input-output tables which document transactions between industrial sectors. This approach is the most practical for answering the questions posed in this study however the results may differ from those using process LCA, where significant processes in the supply chain are individually assessed. Process LCA is commonly used for examining specific supply chains, and estimates of diet-related GHGe using this method tend to be lower [4]. For this reason, the GHGe results reported in this study should not be directly compared to results from other studies where a process LCA approach has been used.

## 5. Conclusions

Notwithstanding the limitations of this modelling, there are some clear synergies in recommendations which advocate a reduction in non-core food consumption and realignment of dietary choices to better conform to Australian Dietary Guidelines. The urgency of climate change, and concern for the environmental sustainability of our food supply, demands informed behaviour changes to reduce diet-related GHGe [36]. This paper takes the first steps in quantifying the GHGe associated with the average Australian diet. The ability to measure GHGe associated with the diet will enable comparisons between changes in dietary intake and GHGe over time. In turn, this data will be useful to inform nutrition and agricultural policy to ensure Australia maintains its quality food supply into the future.
